# Supplementary material for: Prediction of Immunotherapy Response in Hepatocellular Carcinoma Patients Using Pretreatment CT Images
Source: Diagnostics (Basel). 2025 Aug 20;15(16):2090. doi: 10.3390/diagnostics15162090 (PMC12385829; doi:10.3390/diagnostics15162090)
Supplement: Supplementary file 1 [file diagnostics-15-02090-s001.zip › diagnostics-3783590-supplementary.pdf]

Figure S1. Representative axial portal venous phase CT images and segmentation masks from immunotherapy responder and non-responder groups. Each case demonstrates large viable HCC lesion on CT. Despite a similar intrahepatic tumor burden, patients showed different responses to immunotherapy.

(A) *Responder group*: Original CT image (left); Manual segmentation of the entire liver (middle, red); Manual segmentation of viable HCC lesion (right, green).

(B) *Non-responder group*: Original CT image (left); Manual segmentation of the entire liver (middle, red); Manual segmentation of viable HCC lesion (right, green).

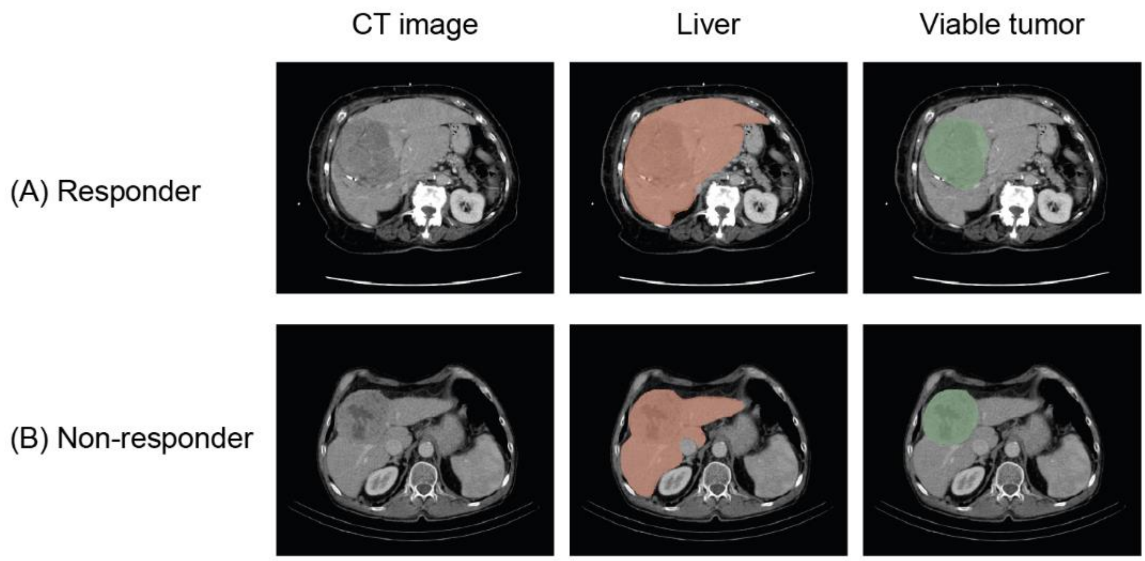

Figure S2 Flow chart of patients

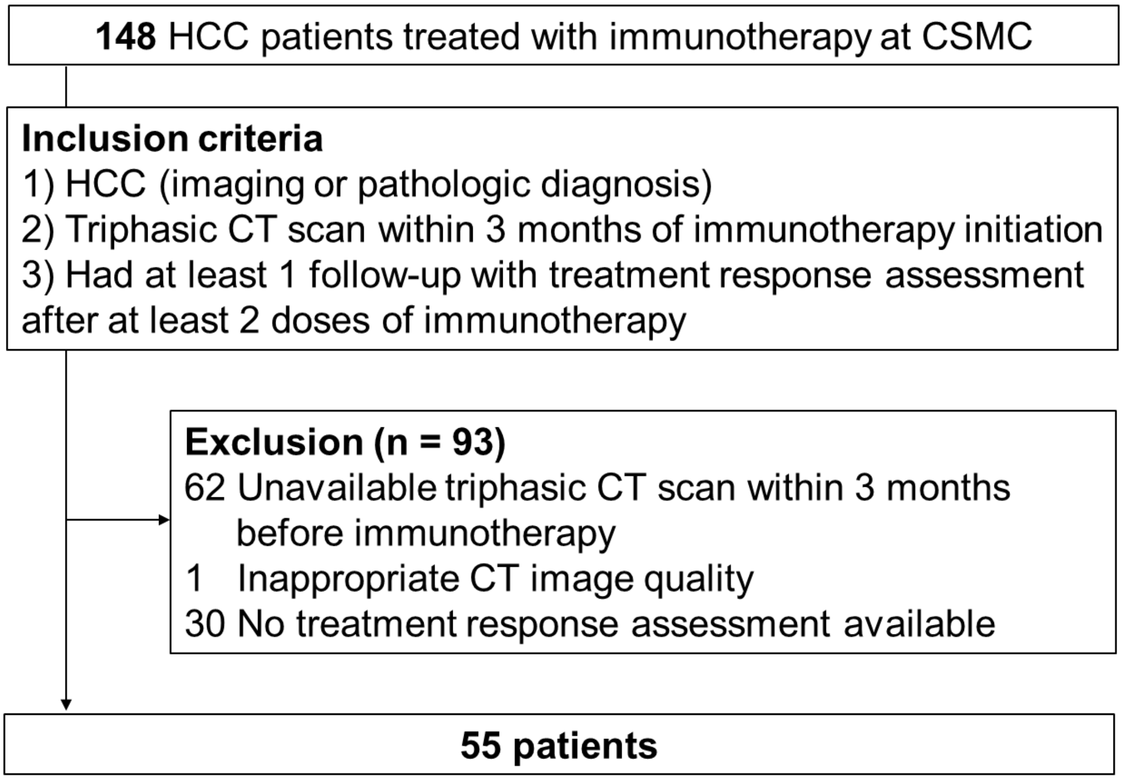

CT, computed tomography; HCC, hepatocellular carcinoma

Table S1 Final selected radiomic features

	Most Predictive Features (n=6)	Additional Common Features (n=4)
Liver	Energy<br>Inverse Gaussian left<br>Inverse Gaussian left focus<br>Gaussian right polar<br>Cluster prominence<br>Cluster shade	Heterogeneity<br>Autocorrelation<br>Homogeneity<br>Entropy
Tumor	Cluster trend<br>Homogeneity<br>Autocorrelation<br>Gaussian<br>Gaussian right focus<br>Gaussian right polar	Energy<br>Left focus<br>Cluster shade<br>Contrast
